# Supplementary material for: Speak to a Protein: An Interactive Multimodal Co-Scientist for Protein Analysis
Source: J Chem Inf Model. 2026 Sep 30;66(19):12391–401. doi: 10.1021/acs.jcim.6c01738 (PMC13663485; doi:10.1021/acs.jcim.6c01738)
Supplement: Supplementary file 1 [file ci6c01738_si_001.pdf]

Supporting Information: Supporting Information for Speak to a Protein: An Interactive Multimodal Co-Scientist for Protein Analysis

Carles Navarro,[*,†,‡] Mariona Torrens,[†] Philipp Tholke,[†] Stefan Doerr,[†] and Gianni de Fabritiis[*,†,‡,¶]

†*Acellera Labs, C/ Doctor Trueta 183, 08005 Barcelona, Spain*

‡*Computational Science Laboratory, Universitat Pompeu Fabra, PRBB, C/ Doctor Aiguader 88, 08003 Barcelona, Spain*

¶*Institució Catalana de Recerca i Estudis Avançats (ICREA), Passeig Lluís Companys 23, 08010 Barcelona, Spain*

E-mail: c.navarro@acellera.com; g.defabritiis@acellera.com

S1 Appendix

S1.1 Tables

Table S1: Fields returned by the literature search tool. Global fields describe the query context; results contain one or more retrieved citations with text passages.

Field	Description / Example Value
`fasta_sequences[]`	One or more FASTA sequences associated with the protein input.
`pdb_ids[]`	PDB identifiers discovered for the input (via cross-references or sequence search). *Example:* ["1H1R", "3DDQ", "7RWE"]
`uniprot_ids[]`	UniProt accessions associated with the input. *Example:* ["P24941"]
`results[].pmcid`	PubMed Central ID for a retrieved article. *Example:* "PMC5291709"
`results[].doi`	Digital Object Identifier for the article. *Example:* "10.7554/eLife.20818"
`results[].content`	Retrieved text passage from the article body (cleaned paragraphs). *Example:* "ATP-binding sites involve Walker-A and -B motifs and a trigger residue that regulates hydrolysis."
`error`	Error message if something went wrong; empty/absent otherwise. *Example:* "Failed to load existing RAG database: ..."

S1.2 Usage Metrics

To demonstrate the practical utility and robustness of the system, we analyzed usage statistics from the public deployment of *Speak to a Protein* between October 2025 and July 2026. These metrics reflect organic adoption by the scientific community. As shown in Figure S1, the platform has seen consistent growth in its user base, reaching 1,921 users of the AI chat. Simultaneously, the volume of interaction has accelerated. Figure S1b displays weekly token consumption, which increased steadily over the monitoring period, peaking at 479 million tokens in June 2026.

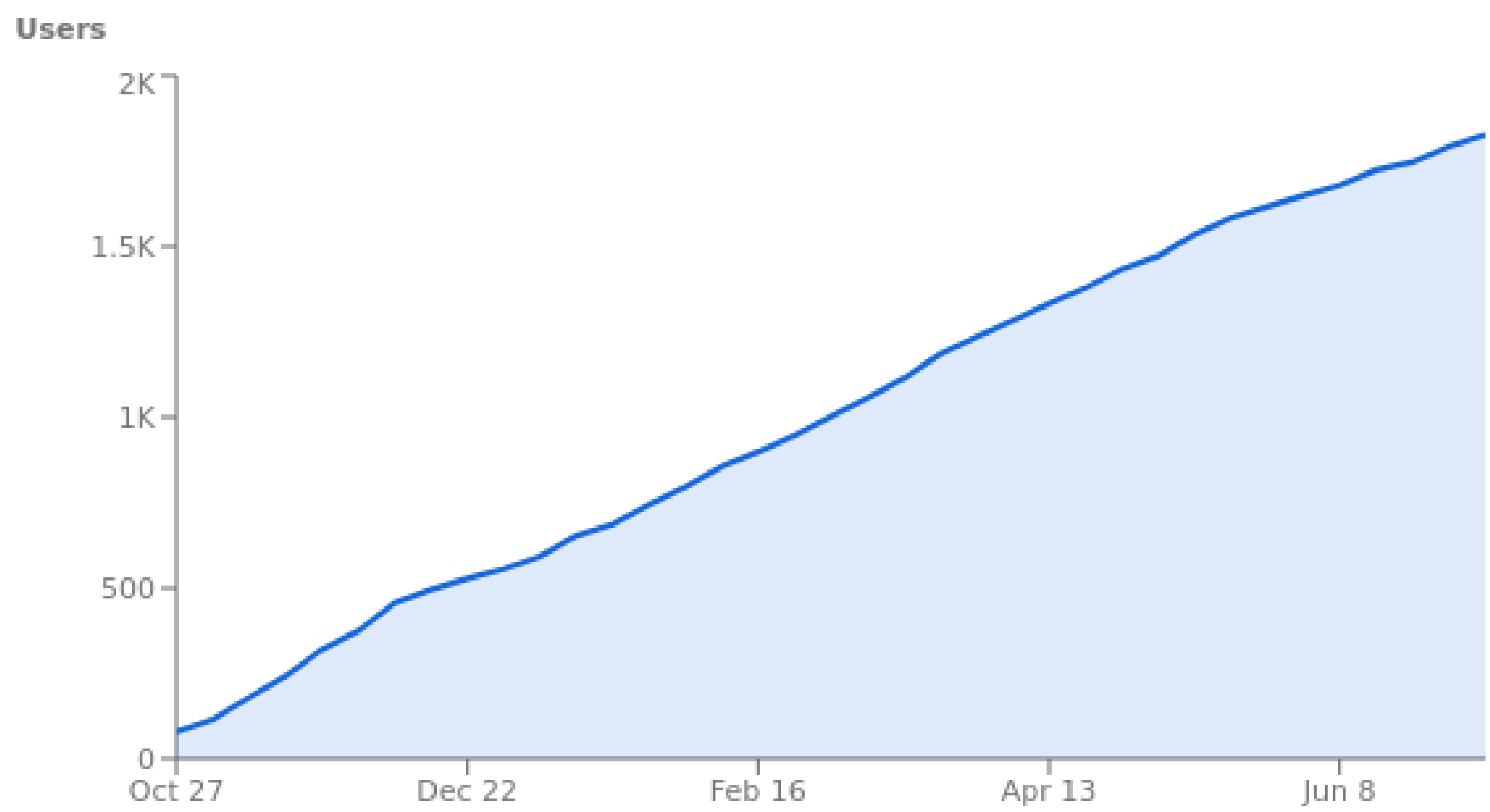

(a) Aggregated number of unique users engaging with the AI Chat.

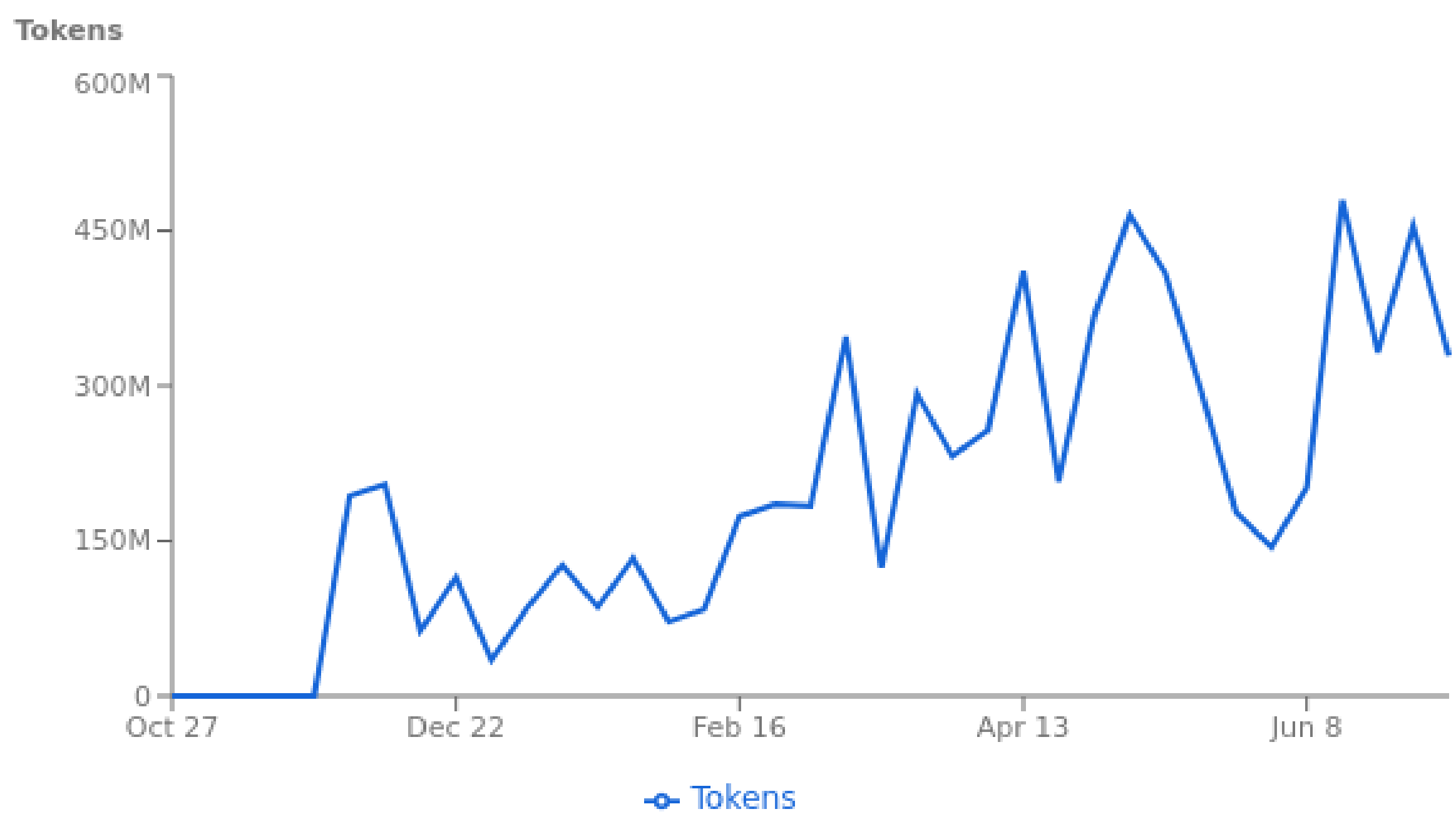

(b) Weekly token consumption across AI Chat sessions.

Figure S1: **Platform Usage Statistics.** Usage metrics from the public release of *Speak to a Protein*, demonstrating steady user adoption and a substantial increase in weekly token consumption from late 2025 through mid-2026.

S1.3 Structural workflow of the D3R/D2R selectivity case study.

Video S1. Full *Speak to a Protein* session for the D3R/D2R selectivity case study, illustrating the three-prompt workflow from structure loading and alignment, through residue-level selectivity analysis, to the assembly of the D3R-selective compound table.

S1.4 Auto-Generated CDK2 Analysis Report

The following pages reproduce the complete report generated by *Speak to a Protein* for the CDK2 case study, exactly as written by the agent from the single prompt shown in Section 4.4 of the main paper. No content has been altered.

CDK2 as a Drug-Discovery Target: Structural, Bioactivity, and Literature-Based Assessment

Speak to a Protein

May 12, 2026

Abstract

CDK2 is a structurally mature and chemically tractable kinase target, but its value is context-dependent rather than universal. To assess CDK2 practically for medicinal chemistry, I integrated the full experimental structure set cross-referenced from UniProt/RCSB, filtered ChEMBL bioactivity, direct binding-site inspection in the 3D viewer, and targeted literature review. The structural evidence is unusually deep: 498 deposited human-CDK2-linked structures, dominated by ligand-bound crystallography, with apo, cyclin-bound, peptide-bound, allosteric, macrocyclic, cryo-EM ternary-complex, and degrader-relevant states. The ATP site is well understood and supports subnanomolar potency, but matched-compound data show that orthosteric selectivity versus other cell-cycle CDKs—especially CDK1 and CDK9—remains difficult. The strongest therapeutic rationale is biomarker-driven: CCNE1-amplified or cyclin-E-high tumors, replication-stress biology, and settings of acquired CDK4/6-inhibitor resistance. The most practical conclusion is that CDK2 is a worthwhile target when paired with patient selection and a modality strategy that addresses selectivity risk. Classical ATP-site programs remain feasible, but allosteric/interface chemistry and targeted degradation appear especially attractive routes to widen therapeutic margin.

1 Executive assessment

Table 1. Practical scorecard for CDK2 as a discovery target.

**Dimension**	Assessment	Practical implication
**Structural tractability**	High	The target is exceptionally well covered by experimental structures across apo, cyclin-bound, orthosteric, allosteric, macrocyclic, and ternary-complex states.
**Biomarker strength**	Moderate–high	The clearest case is not "all-comer" oncology but cyclin-E-driven or CCNE1-amplified disease and tumors that escape CDK4/6 blockade through cyclin E/CDK2 signaling.
**Orthosteric potency**	High	Subnanomolar ATP-site inhibition is routine; the chemistry risk is not potency but selectivity and differentiation.
**Orthosteric selectivity risk**	High	Matched-compound activity shows persistent overlap with CDK1 and other CDKs, limiting the margin for broad ATP-mimetic programs.
**Novel pocket opportunity**	Moderate–high	Two non-ATP opportunities are visible in the structure set: the older ANS site on monomeric CDK2 and a newer cyclin-E-dependent interface pocket.
**Clinical positioning**	Conditional	Best positioned as a biomarker-led program or combination strategy, rather than an unselected monotherapy built only around cell-cycle inhibition.
**Overall verdict**	**Go, but selectively**	CDK2 is attractive when biology, modality, and selectivity plan are aligned; it is less compelling as a generic pan-cancer kinase project.

The core message is straightforward: CDK2 is not short on structural information or potent ligands, but it does require a disciplined translational strategy. If the goal is a best-in-class ATP-site inhibitor, early selectivity pressure against CDK1/CDK4/CDK6/CDK7/CDK9 is essential. If the goal is a differentiated program with a wider therapeutic window, the most interesting opportunities are cyclin-context-dependent allostery and degraders that exploit CDK2-cyclin E biology rather than catalytic inhibition alone.

2 Data sources and scope

This assessment combined four evidence streams: (i) UniProt/RCSB structural metadata for human CDK2 (UniProt P24941), (ii) direct 3D inspection of representative complexes in the Speak to a Protein viewer, (iii) filtered ChEMBL biochemical activity for CDK1/2/4/6/7/9 and CDK2/cyclin complexes, and (iv) targeted literature spanning kinase biology, cyclin-E dependency, allosteric chemistry, and emerging degrader strategies. The quantitative dataset included 498 human-CDK2-linked structures and 3,394 filtered human single-protein CDK2 activity records, plus comparator

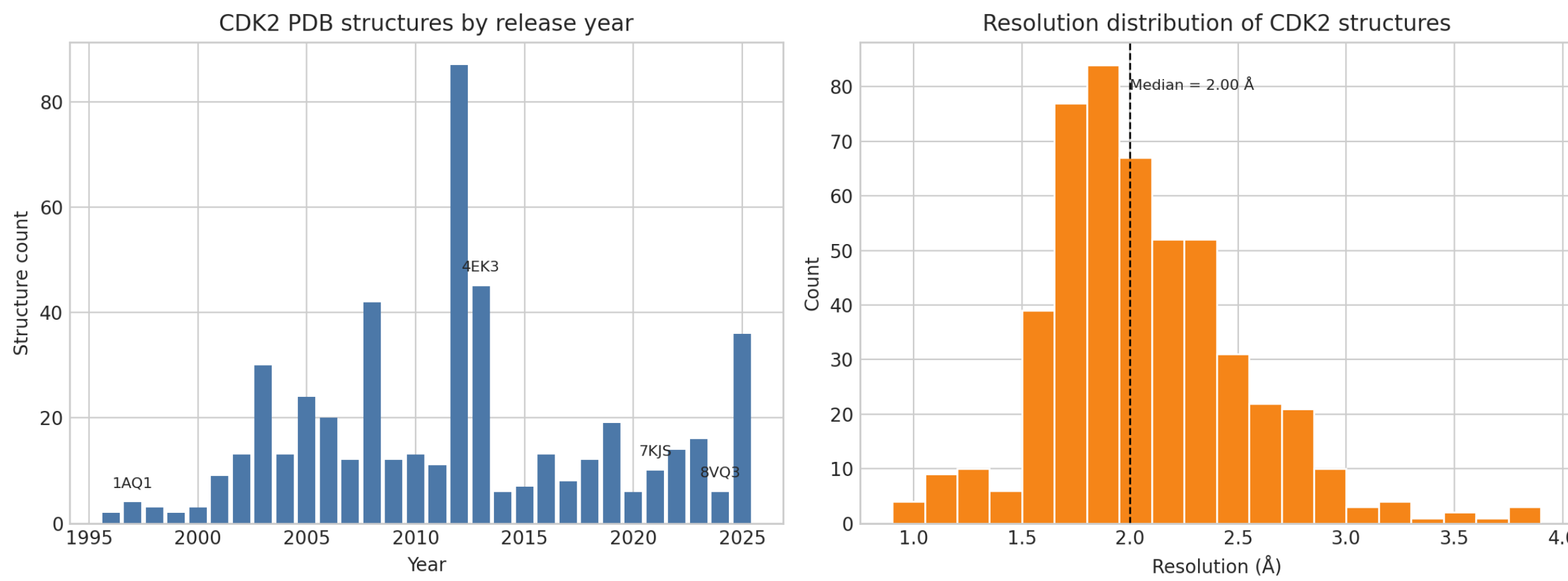

Figure 1. Breadth and quality of the experimental CDK2 structure set. Left: structure deposition has been sustained for nearly three decades, with notable surges during the medicinal-chemistry boom around 2012–2013 and again in the recent wave of selective and allosteric programs. Right: most entries cluster around 1.7 Å to 2.3 Å, enabling confident interpretation of binding modes and local pocket geometry.

datasets for relevant CDKs.

3 Structural landscape: unusually deep for a kinase target

CDK2 is one of the best structurally covered cell-cycle kinases. The current dataset contains 498 linked structures, including 489 X-ray and 9 cryo-EM entries, with a median resolution of 2.0 Å; 467 contain non-polymer ligands and at least 374 are explicitly annotated cyclin complexes. The structural record spans early ATP-site ligands, peptide recruitment-groove complexes, phosphorylated catalytic states, monomeric apo structures, weak allosteric fragments, modern cyclin-E-bound inhibitor complexes, and recent ternary assemblies relevant to degradation.

A useful way to navigate this landscape is to separate structures into three practical buckets:

1. **Activation-state structures**, which explain how cyclin binding reorganizes the kinase and which conformations are relevant to disease-linked cyclin E biology.
2. **Orthosteric ligand complexes**, which define the ATP-site pharmacology and explain why potency is easy but selectivity is hard.
3. **Allosteric or interface complexes**, which offer the best chance of obtaining differentiated selectivity profiles.

Table 2. Representative CDK2 structures worth keeping at the top of a medicinal-chemistry short list.

PDB	State	Resolution (Å)	Why it matters
4EK3	Apo monomer	1.34	Best apo baseline for intrinsic inactive geometry before ligand or cyclin engagement.
1W98	CDK2/cyclin E1	2.15	Canonical structural explanation of cyclin-E-driven activation; especially relevant for cyclin-E-high tumors.
1CKP	Purvalanol B complex	2.05	Classical ATP-site template for hinge-binding CDK2 chemistry.
4KD1	Dinaciclib complex	1.70	Benchmark for potent but broad CDK inhibition.
7KJS	PF-06873600 (ebvaciclib) in CDK2/cyclin E1	2.19	Modern, subnanomolar ATP-site complex linked to CDK2/4/6 translational chemistry.
3PXZ	ANS + orthosteric ligand ternary complex	1.70	Early proof that CDK2 contains a ligandable non-ATP site.
8VQ3	Allosteric interface inhibitor in CDK2/cyclin E1	1.84	Strongest recent structural argument for a selective, cyclin-context-dependent pocket.
8H6P	Selective macrocyclic CDK2/cyclin E1 complex	2.44	Illustrates modern shape-driven selectivity strategies within the ATP site.

3.1 Cyclin binding is the key conformational organizer

The activation-state comparison in Fig. 2 is more informative than a generic "apo versus holo" overlay. In the chosen structures, apo CDK2 (4EK3) lacks the compact, cyclin-stabilized architecture seen in the cyclin E1 complex 1W98. A simple structural marker captures this well: the Lys33–Glu51 catalytic ion pair is open in 4EK3 (17.8 Å minimum NZ–OE distance in the selected structures) but closed in 1W98 (2.7 Å), consistent with cyclin-driven stabilization of the active αC-in state. Notably, the phospho-Thr160 ATP-bound monomer 1B39 still does not look fully cyclin-organized by this measure, reinforcing the practical point that cyclin context is not a decoration around CDK2 biology; it is part of the target state medicinal chemists should care about.

4 Binding-site architecture and what it means for design

4.1 The ATP site is well mapped and pharmacologically forgiving

The orthosteric pocket of CDK2 is highly mature from a structure-based-design perspective. Across five representative inhibitor complexes, the residues most recurrently contacting ligands are Ile10, Ala31, Lys33, Phe80, Glu81, Phe82, Leu83, His84, Asp86, Gln131, Asn132, Leu134, and Asp145. This pattern in Fig. 3 is consistent with decades of kinase chemistry: ligands anchor through the hinge region around Glu81/Leu83, exploit a hydrophobic cavity bounded by Ile10/Val18/Ala31/Phe80,

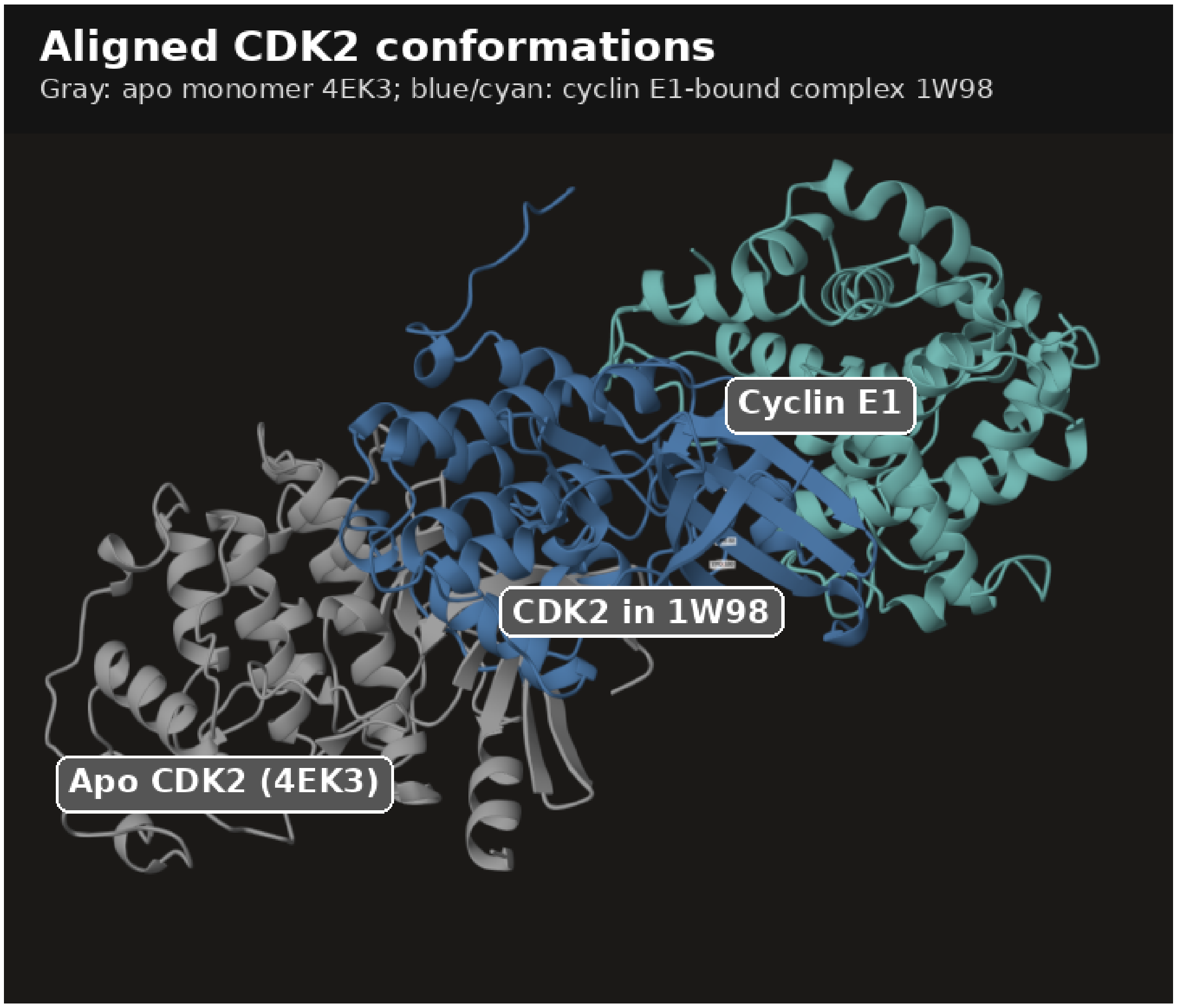

Figure 2. Annotated viewer snapshot of aligned CDK2 conformations. Apo CDK2 (4EK3, gray) is compared with the cyclin E1-bound activated complex (1W98: CDK2 in blue, cyclin E1 in cyan). This is the most useful visual reminder that cyclin binding is a structural event, not just a biochemical assay detail. The cyclin-bound form stabilizes the catalytically competent architecture that medicinal chemistry must usually engage in disease-relevant settings [1].

and often extend toward the solvent-exposed or back-pocket region near Lys89, Gln131, Asn132, and Asp145.

Several practical design lessons follow immediately:

- **Hinge binding is non-negotiable for most ATP-site series.** Classical CDK2 ligands such as purvalanol B (1CKP), dinaciclib (4KD1), and PF-06873600/ebvaciclib (7KJS) all exploit this.
- **Phe80 is a key shape determinant.** Its aromatic bulk helps define the roof of the pocket and limits how much "back-pocket" chemistry can be borrowed from other kinases.
- **The solvent/back-pocket edge is where selectivity attempts usually live.** Gln131, Asn132, Leu134, Lys89, and Asp145 are natural positions for projection and fine-tuning.
- **The site heavily favors type-I-like chemistry.** In practice, the deposited CDK2 set is dominated by active-site ligands that bind the adenine region rather than classical DFG-out type-II motifs.

This is exactly why CDK2 programs can look deceptively easy at the start. The structural playbook for making a potent ATP-site inhibitor is mature. The harder problem is ensuring that

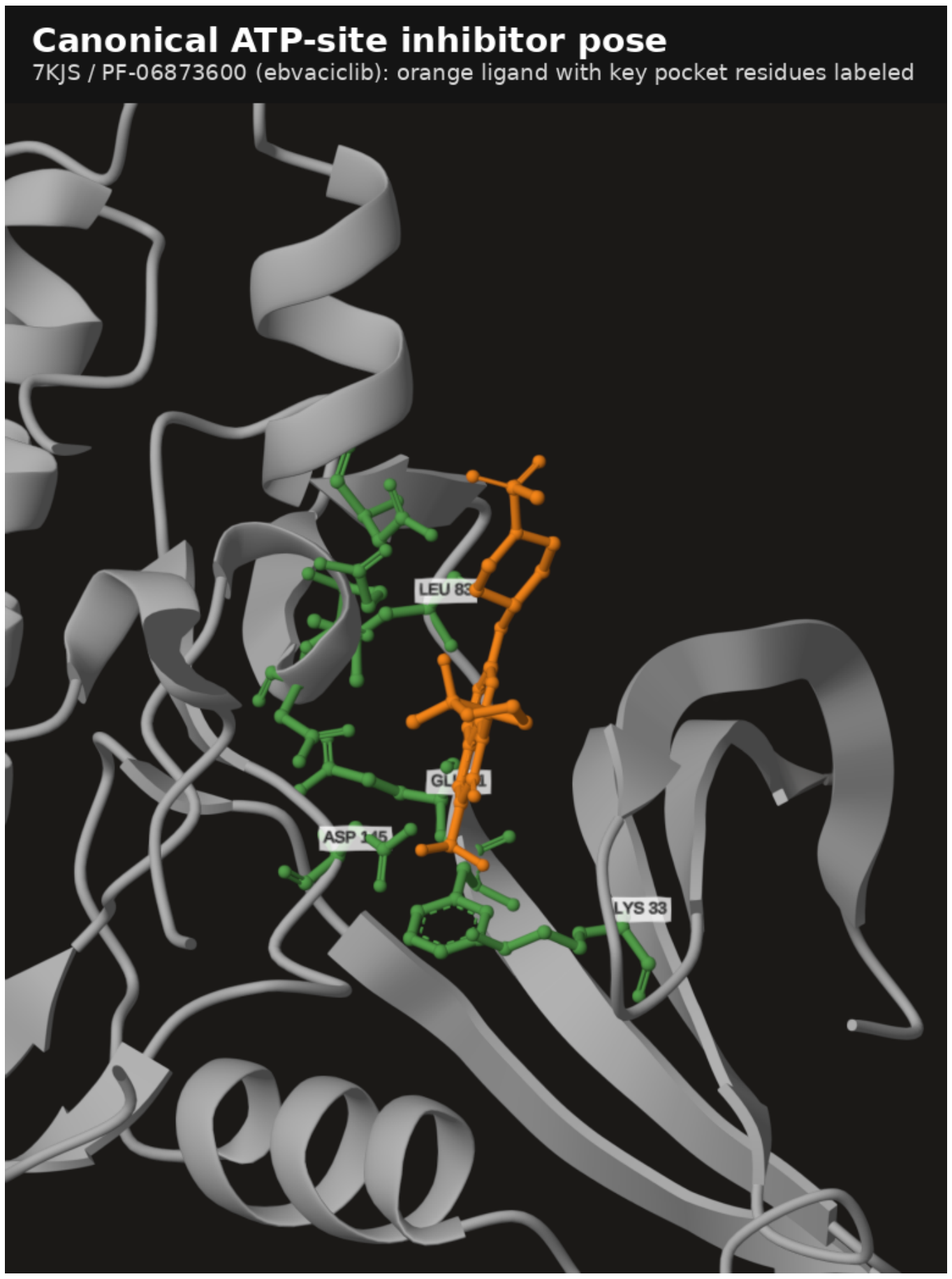

(a) Annotated 3D view of the ATP site in 7KJS.

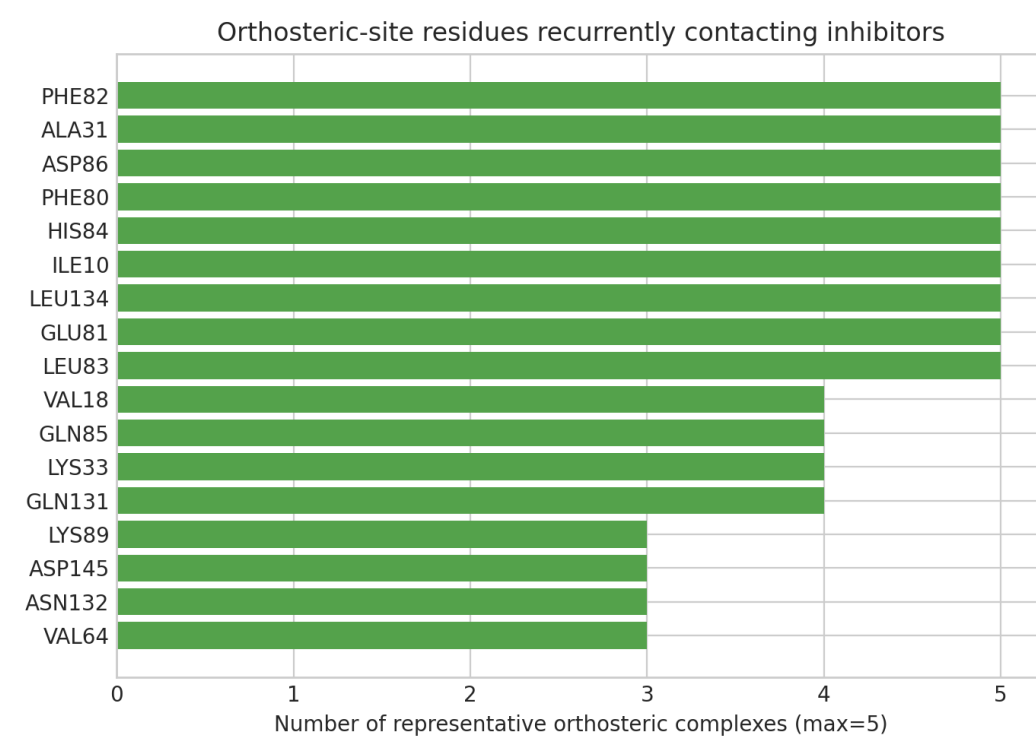

(b) Recurrent contact residues across representative ATP-site complexes.

Figure 3. CDK2 orthosteric architecture. The 7KJS complex shows a modern ATP-site inhibitor pose for PF-06873600 (ebvaciclib), while the contact-frequency analysis highlights the residues medicinal chemists repeatedly use. The site is rich in tractable interactions, which explains why CDK2 potency is easy to achieve; the challenge is not occupancy but clean selectivity.

the same playbook does not deliver a pan-CDK compound.

4.2 Allosteric chemistry is no longer hypothetical

CDK2 has long tempted medicinal chemists with the idea that selectivity might come from binding sites outside the conserved ATP cleft. The first compelling structural evidence came from the ANS-bound complexes reported by Betzi et al. [2], where free CDK2 accommodated 8-anilino-1-naphthalene sulfonate with modest affinity (PDB-linked affinity annotations around 37 μM K_d and 91 μM IC_{50}). Those structures matter because they established ligandability outside the ATP site even though the fragment itself was weak.

The newer interface pocket is more exciting. In 8VQ3, the ligand sits at a cryptic CDK2-cyclin E1 interface site, making simultaneous contact with CDK2 residues such as Arg122, Phe152, Tyr179, and Thr182 and cyclin E1 residues such as Trp95 and Tyr243. That geometry, shown directly in Fig. 4, is medicinally important because it offers a route to selectivity that depends on a protein-protein context absent from many off-target kinases.

For medicinal chemists, the practical implication is clear: if orthosteric selectivity stalls, CDK2

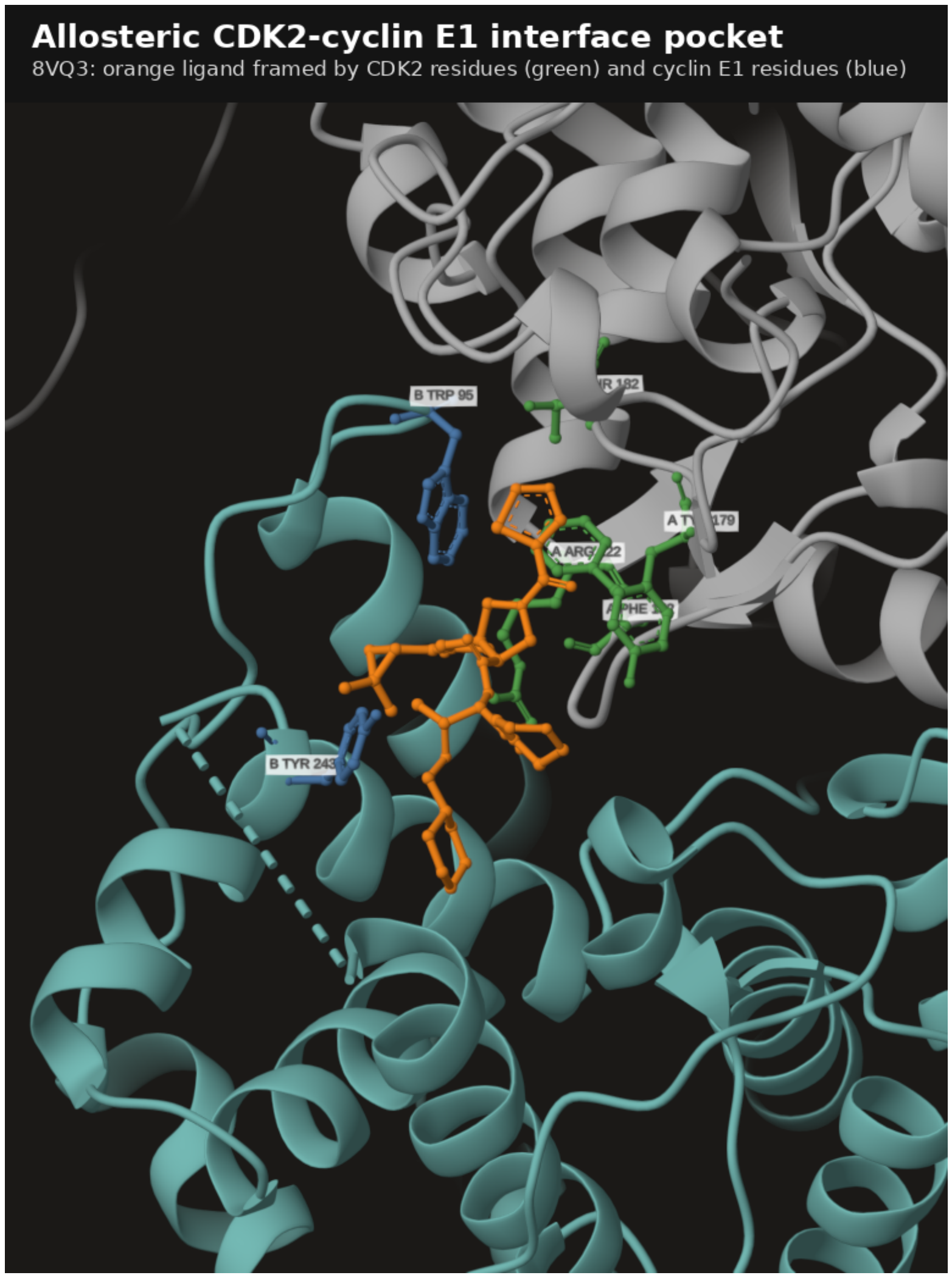

Figure 4. Annotated viewer snapshot of the cyclin-E-dependent allosteric interface pocket in 8VQ3. The orange ligand is framed jointly by CDK2 residues (green) and cyclin E1 residues (blue), illustrating a selectivity concept fundamentally different from classical ATP-site binding. This pocket is the clearest structural justification for differentiated CDK2 chemistry beyond orthosteric hinge binders [2, 3].

is one of the few cell-cycle kinases where a credible structural escape route already exists.

5 Bioactivity: potency is abundant, selectivity is the bottleneck

The filtered ChEMBL dataset contains 3,394 human single-protein CDK2 activity records (2,609 unique molecules) and 2,892 CDK2/cyclin E1 records (2,152 molecules), alongside matched comparator datasets for CDK1, CDK4, CDK6, CDK7, and CDK9. The headline from Fig. 5 is that CDK2 potency is not rare. Median single-protein CDK2 activity is pChEMBL 6.6, and many modern series extend well into the nanomolar and subnanomolar range.

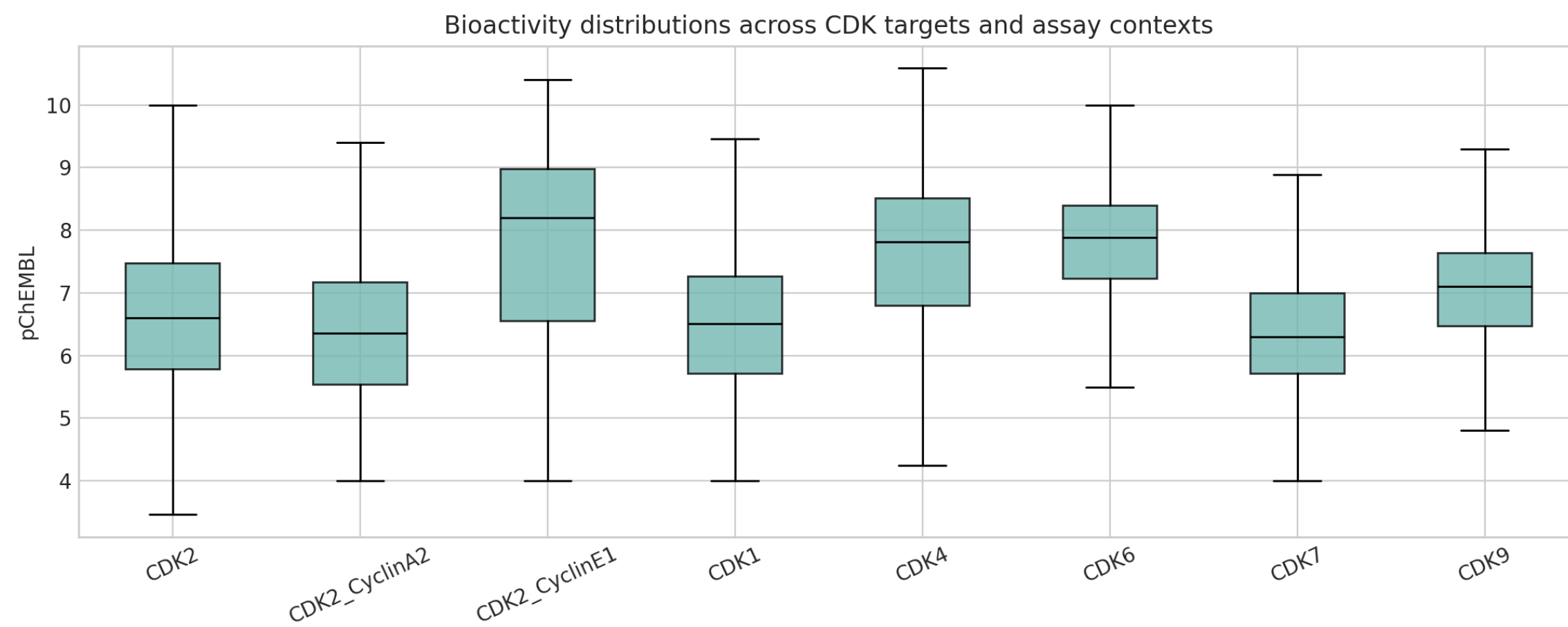

(a) Activity distributions across CDK targets and assay contexts.

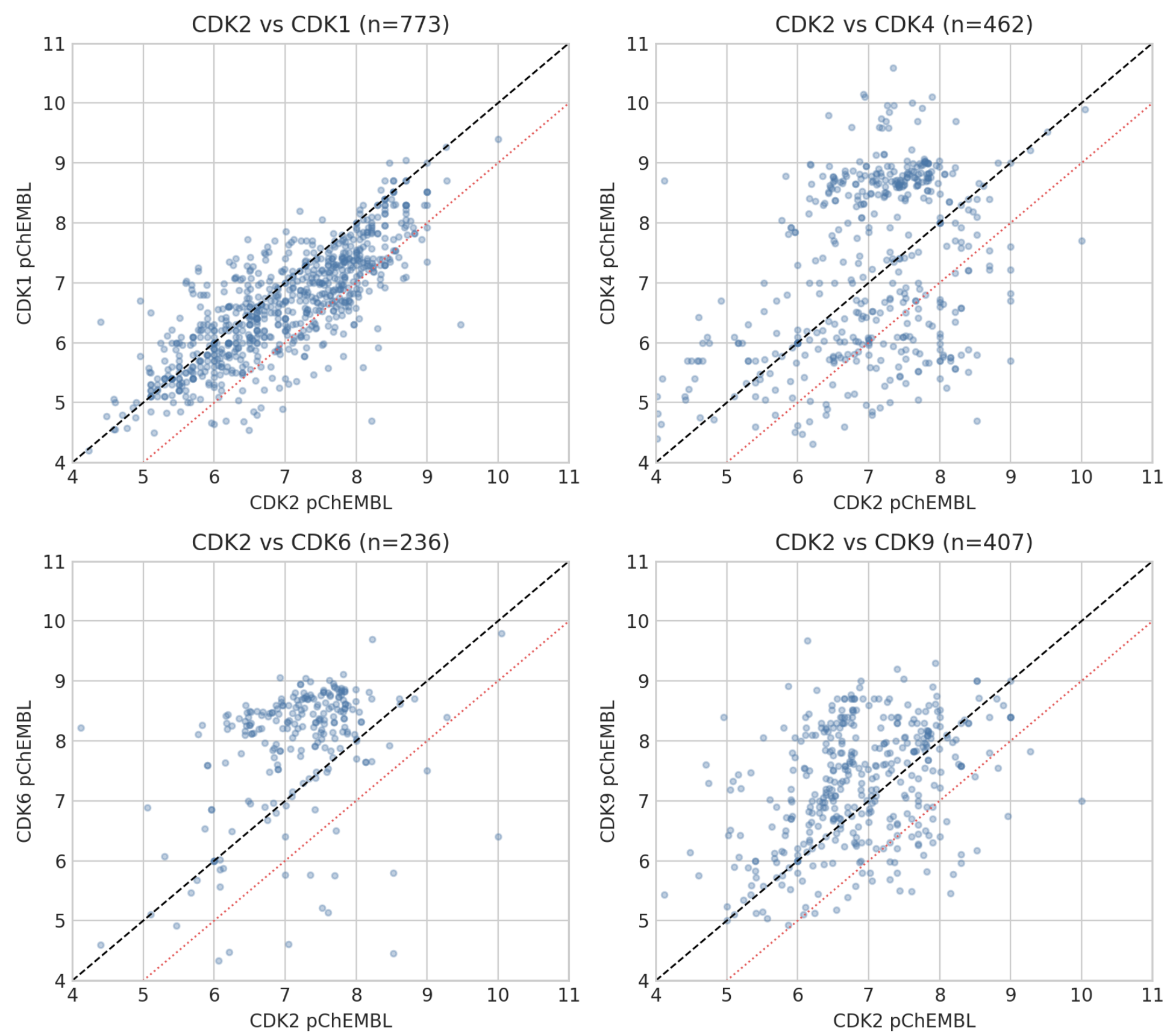

(b) Matched-compound selectivity plots relative to CDK2. The dashed black line is parity; the red dotted line marks a one-log margin in favor of CDK2.

Figure 5. CDK2 potency and selectivity trends from filtered ChEMBL activities. The elevated CDK2/cyclin E1 potency distribution likely reflects enrichment for recent optimized campaigns rather than a directly comparable baseline shift in intrinsic targetability. Across matched molecules, CDK2 selectivity over CDK1 is achievable but uncommon, while overlap with CDK9 is also frequent.

Three trends matter most:

Table 3. Named inhibitors that are useful reference points for CDK2 programs. Values are maximum filtered pChEMBL observations per target from the ChEMBL-derived dataset.

Compound	CDK1	CDK2	CDK4	CDK6	CDK7	CDK9
Ebvaciclib (PF-06873600)	–	10.05	9.89	9.80	–	–
Staurosporine	9.40	10.00	7.70	6.40	7.35	7.00
Dinaciclib	9.00	9.00	9.00	7.50	7.38	9.00
Milciclib	7.35	7.35	7.35	5.76	6.54	–
Seliciclib	7.09	7.05	4.79	4.61	7.37	7.37

1. Orthosteric potency is solved. Representative benchmarks make this obvious. Purvalanol B in 1CKP is a low-nanomolar CDK2 inhibitor (about 6 nM in the filtered ChEMBL set), dinaciclib reaches roughly 1 nM, and PF-06873600/ebvaciclib is subnanomolar in the CDK2/cyclin E structure 7KJS. Staurosporine is even more potent, but as a classic promiscuous kinase ligand it illustrates the wrong lesson: exceptional potency does not equal useful selectivity.

2. Selectivity over CDK1 remains the hardest medicinal-chemistry test. Among compounds measured on both CDK2 and CDK1, the median CDK2 advantage is only 0.32 pChEMBL units, and just 15.3% achieve a full one-log selectivity margin in favor of CDK2. That is a manageable problem, not an impossible one, but it is a real one.

3. Many "potent CDK2" ligands are still broad cell-cycle kinase inhibitors. Matched benchmark compounds make this concrete:

Ebvaciclib and dinaciclib show how easy it is to make potent, clinically relevant CDK2 binders that still cover other G1 or transcriptional CDKs. Seliciclib shows the opposite tradeoff: a somewhat more differentiated profile, but at much lower potency. This is the central chemistry tension of CDK2.

One caution is important. The CDK2/cyclin E1 dataset appears more potent overall than the monomeric CDK2 dataset. I do *not* interpret that as proof that cyclin E1 complexes are intrinsically easier to inhibit. The more likely explanation is series bias: the cyclin-E dataset is enriched for modern, structure-guided, biomarker-focused campaigns and thus for stronger compounds.

6 What the literature says about CDK2 as a therapeutic target

The literature argues against a naive, one-size-fits-all view of CDK2. On the one hand, CDK2 is not universally essential for mammalian proliferation. Genetic evidence shows that Cdk1 can substitute for the interphase CDKs in surprisingly broad settings, which helps explain why simple "cell-cycle shutdown" logic has not been enough to validate CDK2 as a universal oncology target [4]. This redundancy is a real biological risk and should temper enthusiasm for unselected patient populations.

On the other hand, the same literature points to clear *context-specific* vulnerabilities:

- **CCNE1-amplified high-grade serous ovarian cancer.** Multiple studies support CDK2 dependence in this setting and show that CDK2 suppression or inhibition is especially relevant when cyclin E1 is amplified or overexpressed [5–7].

- **Replication-stress biology.** In CCNE1-amplified ovarian models, CDK2 supports homologous-recombination-mediated repair of collapsed replication forks, implying rational combinations with DNA-damaging agents rather than pure single-agent use [7].
- **CDK4/6-inhibitor resistance.** Preclinical and translational work around PF-06873600 supports the idea that cyclin E/CDK2 signaling becomes an escape route when CDK4/6 blockade is insufficient, especially in breast cancer models [8, 9].
- **Targeted degradation.** Recent degraders that co-deplete CDK2 and cyclin E appear particularly interesting because they address both catalytic signaling and the cyclin-E-driven state itself, while improving selectivity over CDK1 [10].

The literature therefore supports a nuanced but encouraging conclusion: CDK2 is not the best target for a broad "all tumors need this kinase" thesis, but it is increasingly well supported in biomarker-defined and resistance-defined oncology niches.

7 Recommended next steps for medicinal chemistry

7.1 If the goal is an ATP-site small-molecule program

Use 1CKP, 4KD1, 7KJS, and 8H6P as the core structural set. They cover classical hinge-binding, pan-CDK risk, modern cyclin-E-complex binding, and selective macrocyclic shape control. The required discipline is early selectivity triage:

1. Profile every design cycle against at least CDK1, CDK4, CDK6, CDK7, and CDK9, not just CDK2.
2. Exploit the Gln131/Asn132/Leu134/Asp145 edge for projection and shape differentiation.
3. Treat Phe80 as a design constraint: useful for sculpting fit, but also a source of steric convergence across CDK chemotypes.
4. Avoid congratulating the program too early for potency; subnanomolar CDK2 alone is not a differentiator.

7.2 If the goal is a differentiated selectivity strategy

Prioritize the allosteric/interface pocket represented by 8VQ3 and 8VQ4, with 3PXZ retained as historical proof of ligandability. This route is attractive because the pocket depends on cyclin context and therefore encodes information absent from the ATP site. The medicinal-chemistry challenge is greater, but the reward is better odds of escaping the pan-CDK trap.

7.3 If the goal is translational advantage rather than pure enzymology

A degrader or molecular-glue-like strategy deserves serious consideration. The most persuasive biology in the recent literature comes from co-targeting the CDK2-cyclin E state rather than just reducing ATP turnover [10]. Structurally, the appearance of cryo-EM ternary complexes in the PDB means this is no longer a purely conceptual modality for CDK2.

7.4 Biology package to run alongside chemistry

For decision quality, the chemistry program should be paired from the start with:

- CCNE1-amplified ovarian cancer models,
- cyclin-E-high or CDK4/6-inhibitor-resistant breast cancer models,
- RB phosphorylation and S-phase/replication-stress pharmacodynamic readouts,
- combination studies with DNA-damaging agents in replication-stress-driven settings,
- a clear benchmark against whether catalytic inhibition alone outperforms or underperforms degradation.

8 Bottom line

CDK2 is a good drug-discovery target when asked the right question. It is *not* best framed as a universally essential cell-cycle kinase waiting for a better inhibitor. It *is* a highly tractable, structurally rich, and biologically plausible target in cyclin-E-driven tumors, replication-stress states, and CDK4/6-resistance settings. The structure set is already strong enough to support serious medicinal chemistry, and the literature now supports differentiated strategies beyond classical ATP competition.

If I were prioritizing a portfolio, I would rank CDK2 as follows:

1. **Highest priority:** biomarker-led programs in CCNE1-amplified or cyclin-E-high disease, especially if allosteric or degrader modalities are feasible.
2. **Medium priority:** orthosteric ATP-site chemistry when there is a strong profiling plan and a clear reason to believe a therapeutic window can emerge despite CDK overlap.
3. **Lowest priority:** undifferentiated broad-usage CDK2 monotherapy concepts without biomarker selection or a strategy for selectivity versus other CDKs.

In short: the evidence supports CDK2 as a *practical* target, but only for programs that treat selectivity and patient selection as first-class design variables.

References

[1] R. Honda, E. D. Lowe, E. Dubinina, V. Skamnaki, A. Cook, N. R. Brown, et al. The structure of cyclin e1/cdk2: implications for cdk2 activation and cdk2-independent roles. *EMBO Journal*, 2005. doi: 10.1038/sj.emboj.7600554.

[2] S. Betzi, R. Alam, M. Martin, D. J. Lubbers, H. Han, S. R. Jakkaraj, et al. Discovery of a potential allosteric ligand binding site in cdk2. *ACS Chemical Biology*, 2011. doi: 10.1021/cb100410m.

[3] Y. Zhang, Z. Liu, M. Hirschi, O. Brodsky, E. Johnson, S. J. Won, et al. An allosteric cyclin e-cdk2 site mapped by paralog hopping with covalent probes. *Nature Chemical Biology*, 2025. doi: 10.1038/s41589-024-01738-7.

[4] D. Santamaria, C. Barriere, A. Cerqueira, S. Hunt, C. Tardy, K. Newton, et al. Cdk1 is sufficient to drive the mammalian cell cycle. *Nature*, 2007. doi: 10.1038/nature06046.

[5] G. Au-Yeung, F. Lang, W. J. Azar, C. Mitchell, K. E. Jarman, K. Lackovic, et al. Selective targeting of cyclin e1-amplified high-grade serous ovarian cancer by cyclin-dependent kinase 2 and akt inhibition. *Clinical Cancer Research*, 2017. doi: 10.1158/1078-0432.CCR-16-0620.

[6] D. Etemadmoghadam, G. Au-Yeung, M. Wall, C. Mitchell, M. Kansara, E. Loehrer, et al. Resistance to cdk2 inhibitors is associated with selection of polyploid cells in ccne1-amplified ovarian cancer. *Clinical Cancer Research*, 2013. doi: 10.1158/1078-0432.CCR-13-1337.

[7] V. E. Brown, S. L. Moore, M. Chen, N. House, P. Ramsden, H. J. Wu, et al. Cdk2 regulates collapsed replication fork repair in ccne1-amplified ovarian cancer cells via homologous recombination. *NAR Cancer*, 2023. doi: 10.1093/narcan/zcad039.

[8] K. D. Freeman-Cook, R. L. Hoffman, D. C. Behenna, B. Boras, J. Carelli, W. Diehl, et al. Discovery of pf-06873600, a cdk2/4/6 inhibitor for the treatment of cancer. *Journal of Medicinal Chemistry*, 2021. doi: 10.1021/acs.jmedchem.1c00159.

[9] K. Freeman-Cook, R. L. Hoffman, N. Miller, J. Almaden, J. Chionis, Q. Zhang, et al. Expanding control of the tumor cell cycle with a cdk2/4/6 inhibitor. *Cancer Cell*, 2021. doi: 10.1016/j.ccell.2021.08.009.

[10] N. Kwiatkowski, T. Liang, Z. Sha, P. N. Collier, A. Yang, M. Sathappa, et al. Cdk2 heterobifunctional degraders co-degrade cdk2 and cyclin e resulting in efficacy in ccne1-amplified and overexpressed cancers. *Cell Chemical Biology*, 2025. doi: 10.1016/j.chembiol.2025.03.006.

S1.5 Reliability of the agent's case studies

Every verifiable claim of the two case studies was checked against its primary source: 75 claim entries that resolve into the 136 individual statements of Table S2, each one a number, a name or an assignment. Judgements were left out, because they have no ground truth. Where an independent route existed we took it rather than reuse the agent's own tools: coordinates were read with a parser written for this check, the D3R and D2R positions were matched through the GPCRdb generic numbering instead of the alignment the session computed, and every reference was resolved through Crossref and Europe PMC. Both databases moved after the sessions ran, so each query is anchored to the state the agent saw, version 275 of UniProt entry P24941, which lists the 498 structures the agent retrieved where the entry today lists 512, and release 36 of ChEMBL, which the web service still served on both session dates. Of the 136 statements, 135 are correct. The one error is the number of cyclin complexes, given as at least 374 where the correct number is 166: the session counted every entry whose chain description contains the word *cyclin*, and that word also appears in *Cyclin-dependent kinase 2*, the name of the kinase itself, so 208 of the 374 entries carry no cyclin chain. The claim files, the scripts and the evidence retrieved for each claim are released with the paper.

Table S2: Verifiable statements of the two case studies, checked against their primary sources at the database state each session saw.

**Case study**	**Category**	**Statements**	**Reproduced**
CDK2	Structure counts and resolution statistics	8	7
CDK2	Identity, state and resolution of a named entry	9	9
CDK2	Distances and contacts from coordinates	34	34
CDK2	Counts and statistics over the activity export	8	8
CDK2	Potency of a named compound	35	35
CDK2	Compound and ligand identity	2	2
CDK2	References resolved and supporting their statement	12	12
D3R	Identity and state of a named entry	3	3
D3R	Residue assignments and generic numbering	7	7
D3R	Potency values and selectivity ratios	11	11
D3R	Compound scaffold assignments	7	7
**Total**		**136**	**135**
